# A mechanism underlying the association of A*β* plaque and lipid droplets in Alzheimer's disease

**DOI:** 10.1016/j.apsb.2025.06.018

**Published:** 2025-06-24

**Authors:** Lixuan Ren, Xiwen Ma, Jianping Ye

**Affiliations:** a Institute of Trauma and Metabolism, Zhengzhou Central Hospital Affiliated to Zhengzhou University, Zhengzhou 450007, China; b Tianjian Laboratory of Advanced Biomedical Sciences, Academy of Medical Sciences, Zhengzhou University, Zhengzhou 450001, China

**Keywords:** A*β*, Microglia, Lipid droplet, Alzheimer's disease, DGAT2

The mechanism of lipid droplet (LD) formation in glial cells is an active area in the field of Alzheimer's disease (AD) pathology. A recent study titled “Amyloid-*β* induces lipid droplet-mediated microglial dysfunction *via* the enzyme DGAT2 in Alzheimer's disease” by Prakash et al.^1^ is published in *Immunity* to report a role of A*β* protein in the induction of LD formation in microglia for the phagocytosis dysfunction in AD. In this context, diacylglycerol *O*-acyltransferase 2 (DGAT2) is identified as a potential therapeutic target for its role in LD formation.

AD is characterized by amyloid-*β* (A*β*) plaque accumulation as a result of microglia failure in clearance of the plaques. Although microglial dysfunction in phagocytosis is known to contribute to A*β* plaque formation, the underlying mechanisms remain unclear for the phagocytosis defect. LD accumulation in microglia is a pathological character of AD, which was first reported by Dr. Alzheimer together with the A*β* plaques in the brains of AD patients. However, the significance of LD accumulation was largely overlooked in the AD field for nearly a century, until recent findings identified the apolipoprotein APOE4 as a genetic risk factor for AD. Microglia with LD accumulation and transition into a dysfunctional state are known as lipid droplet-accumulating microglia (LDAM). LDAM are increased in AD brains and enriched in individuals with the APOE4/4 genotype. Emerging research has uncovered a connection between genetic risk factors and accumulation of LDs in microglia, which results in production of neurotoxic factors from the microglia^2^^,^^3^. APOE4 increases LD formation by interrupting lipid metabolism, and thereby impairing neuronal function in the mechanism of cognitive decline^4^^,^^5^. The new study demonstrates that A*β* promotes LD formation by induction of lipid-synthesizing enzyme DGAT2, which in turn impairs the phagocytic activity of microglia.

The study first revealed that LD accumulation was increased in microglia of 5×FAD mice, in which A*β* accumulation is increased at 2–3 months of age by the transgene, and cognitive function is impaired after 6 months in age^6^. LD accumulation occurred after A*β* accumulation at 5–7 months of age. The degree of accumulation was significantly influenced by age, sex, and brain region. The observation was made by comparing the neutral lipid content in microglia isolated from the mouse brain using flow cytometry. The AD mice exhibited a substantial increase in cerebral LDs at 5–7 months of age, with female mice showing a greater increase. The difference was not observed in younger mice at 3–4 months of age. The data suggest that LD accumulation occurs after A*β* deposition in the 5×FAD mice.

The distribution of LD was investigated in multiple brain regions using label-free stimulated Raman scattering (SRS) microscopy. Hippocampal sections showed a higher LD burden in 5×FAD female mice compared to wild-type (WT) controls, with numerous LDs distribution around A*β* plaques. This observation was confirmed by immunohistochemical co-staining (methoxy X04 for amyloid plaques, LipidTox for lipids, IBA1 for microglia). Similar patterns were observed in the cortex and hippocampal subfields (CA1 and subiculum) of 5×FAD females. Across these regions, LD ^+^ microglia were located mostly around A*β* plaques. In the subiculum, microglia adjacent to plaques demonstrated an amoeboid morphology with shorter processes. Correspondingly, LD^+^ microglia were mostly clustered nearby the plaques, with density declining away from the plaque cores. A parallel analysis of postmortem hippocampal tissue of AD patients demonstrated a significantly higher LD density compared to the non-AD individuals. The LD-laden microglia were substantially elevated in the hippocampi of AD patients, consistent with the findings in the mouse model.

Microglial phagocytosis of A*β* is a critical mechanism for plaque clearance^7^. To investigate whether direct exposure to A*β* would impair the phagocytic function in microglia, microglia were isolated from the mice and treated in cell culture with A*β*^pH^—a pH-dependent fluorescent probe that emits green fluorescence in acidic lysosomes upon phagocytosis. Subsequently, the cells were analyzed using flow cytometry. The results showed that although both 5×FAD and WT microglia exhibited an increase in LD content in response to the A*β* exposure, only the 5×FAD microglia exhibited a reduction in phagocytic activity (by 40%).

To investigate whether A*β* directly induces the LD formation, the WT microglia were treated with A*β* in cell culture for varying durations at 1, 12, and 24 h. All intracellular and extracellular lipids were quantified and characterized by mass spectrometry-based multiple reaction monitoring (MRM) profiling. After 1 h of treatment, the free fatty acids (FFAs) showed the most significant changes, particularly very-long-chain saturated FFAs such as C20:0, C22:0, and C19:0. However, when the treatment duration reached 24 h, triglycerides (TGs) exhibited the most notable changes instead of FFAs. Overall, FFAs initially increased at the beginning, while TG levels rose at 24 h. Pathway analysis revealed that A*β* activated the glycerol phosphate and monoacylglycerol pathways, promoting TG synthesis. This metabolic reprogramming was associated with a reduction in phagocytic capacity in the microglia. These findings indicate that A*β* protein directly promotes the formation of LDs in microglia by inducing lipogenesis.

At the molecular level, the genes underlying the A*β*-induced lipogenesis were investigated. DGAT2 was identified as a key enzyme activated by A*β* exposure in the induction of LD accumulation. DGAT2 is a metabolic enzyme that catalyzes the conversion of diacylglycerol (DAG) into TGs in the cytosol. Elevated levels of DGAT2 protein were observed in LD-laden microglia in both 5×FAD mouse brains and AD patient brains. Inhibition of DGAT2 (D2i) reduced LD formation and restored phagocytic function of microglia in A*β* clearance. To suppress the DGAT2 activity *in vivo*, a protein degrader targeting DGAT2 was infused into the brain at lateral ventricles of 11- to 24-month-old 5×FAD mice. Animals that received the drug for a period of 1 week showed a significant reduction in the A*β* plaque burden, which was observed by comparing the treated and untreated groups. The plaque reduction was approximately 51% in the subiculum region of the hippocampus and LD reduction was 40% in LD-positive microglia near plaques.

This study proposed a molecular model, in which A*β* exposure leads to the accumulation of FFAs in microglia, and DGAT2 converts the FFAs into TGs, resulting in LD formation. This metabolic rewiring impairs phagocytic activity of microglia, promoting the A*β* plaque accumulation. The study also explored the therapeutic potential of targeting DGAT2. Inhibition of DGAT2, either through gene knockdown or protein degradation, showed promise in restoring microglial function and reducing plaque burden—even at advanced stages of AD. However, since DGAT2 is expressed in various cell types, systemic inhibition could lead to serious side effects. Cell type-specific degradation of DGAT2 in the brain, as demonstrated in preclinical models, may effectively avoid the systemic complications.

The study has several limitations. First, the impact of the findings on cognitive function was not assessed in the mouse model, which limits the overall significance of the results. Second, other potential molecules involved in LD formation were not investigated. Third, the mechanism of phagocytosis inhibition by LD was not investigated. Additionally, the mechanisms underlying the FFA induction by A*β* exposure, as well as the effects on other cell types, remain unexplored.

In conclusion, this study demonstrates that A*β* induces LD accumulation in microglia by promoting triglyceride synthesis through DGAT2 activation. The resulting LD buildup impairs microglial phagocytic function, thereby exacerbating A*β* plaque formation in AD. Inhibiting DGAT2 activity may represent a promising therapeutic strategy to restore microglial function in A*β* clearance, ultimately reducing plaque deposition in the brain (Fig. 1).

**Figure 1:**
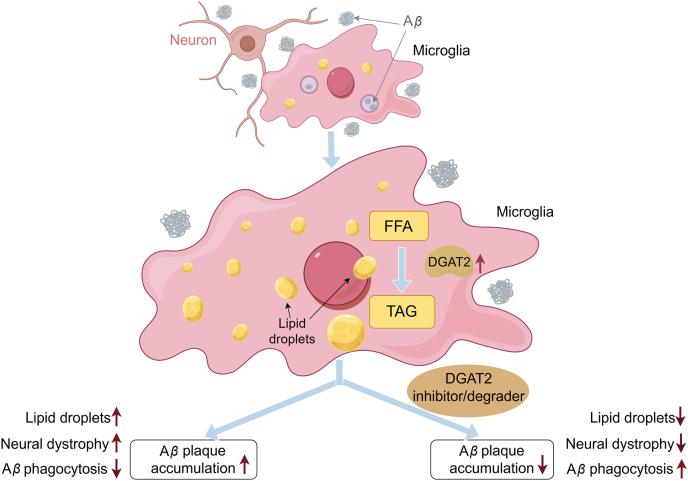
Pathway of A*β* induction of lipid droplets in microglia.

## Author contributions

Lixuan Ren drafted the manuscript. Xiwen Ma and Jianping Ye provided the idea and revised the manuscript.

## Conflicts of interest

The authors declare no conflicts of interest.
